# Opa1 Is Required for Proper Mitochondrial Metabolism in Early Development

**DOI:** 10.1371/journal.pone.0059218

**Published:** 2013-03-14

**Authors:** Jennifer J. Rahn, Krista D. Stackley, Sherine S. L. Chan

**Affiliations:** Department of Drug Discovery and Biomedical Sciences, Medical University of South Carolina, Charleston, South Carolina, United States of America; National Institutes of Health, United States of America

## Abstract

Opa1 catalyzes fusion of inner mitochondrial membranes and formation of the cristae. *OPA1* mutations in humans lead to autosomal dominant optic atrophy. OPA1 knockout mice lose viability around embryonic day 9 from unknown reasons, indicating that OPA1 is essential for embryonic development. Zebrafish are an attractive model for studying vertebrate development and have been used for many years to describe developmental events that are difficult or impractical to view in mammalian models. In this study, Opa1 was successfully depleted in zebrafish embryos using antisense morpholinos, which resulted in disrupted mitochondrial morphology. Phenotypically, these embryos exhibited abnormal blood circulation and heart defects, as well as small eyes and small pectoral fin buds. Additionally, startle response was reduced and locomotor activity was impaired. Furthermore, Opa1 depletion caused bioenergetic defects, without impairing mitochondrial efficiency. In response to mitochondrial dysfunction, a transient upregulation of the master regulator of mitochondrial biogenesis, *pgc1a*, was observed. These results not only reveal a new Opa1-associated phenotype in a vertebrate model system, but also further elucidates the absolute requirement of Opa1 for successful vertebrate development.

## Introduction

Mitochondria are dynamic organelles, and undergo fission, fusion and replication/biogenesis in response to energy demands and stress [1], [2]. *OPA1* encodes a dynamin-related GTPase targeted to the inner mitochondrial membrane and has been demonstrated to play critical roles in mitochondrial fusion, cristae remodeling, and sequestration and release of cytochrome c [3]. In humans, the OPA1 protein exists as 6–9 isoforms that are generated by alternate splicing between exons 4, 4b, and 5b and/or proteolytic processing [4]. Some OPA1 isoforms are differentially located within the mitochondrial intermembrane space, and the various isoforms may facilitate division of labor for the many roles OPA1 plays in the cell [4]. *In vitro* studies have demonstrated the importance of OPA1 in mitochondrial form and function, as down-regulation of *OPA1* leads to disruption of inner mitochondrial membrane fusion in addition to impaired respiration (bioenergetics) and loss of mitochondrial membrane potential [5], [6]. Additionally, *OPA1* has a role in maintaining mitochondrial DNA (mtDNA) stability and integrity by impacting genome mixing that occurs during mitochondrial fusion [7].

Mutations in *OPA1* are associated with autosomal dominant optic atrophy (ADOA) in humans, a disease characterized by progressive loss of visual acuity, desensitization of central visual field, optic nerve pallor, and eventual blindness [8], [9]. Histologically, advanced stages of the disease are characterized by selective loss of the retinal ganglion cell (RGC) layer and ascending atrophy of the optic nerve [9]. To date, over 200 pathogenic mutations have been identified in *OPA1* ranging in location throughout the coding sequence with the exception of exons 4, 4b and 5, and include substitutions, deletions, and insertions [10]. Haploinsufficiency appears to play a major role in pathogenicity of ADOA suggesting that homozygous mutations may be embryonic lethal [10]. As with other diseases associated with mutations in mitochondrial genes, disease severity and age of onset appear to vary even within family members bearing the same mutation [11]. Up to 20% of patients bearing *OPA1* mutations develop additional phenotypes including deafness, progressive external ophthalmoplegia, myopathy, and neuromuscular complications. This more severe set of phenotypes is often referred to as “OPA1-plus” [12], and is variably associated with mtDNA mutations and deletions as well as mtDNA depletion [13] as is often noted in patients with other mitochondrial diseases [11], [13], [14].

Two heterozygous mouse models of OPA1 have been developed to explore the disease characteristics of ADOA. One model introduces a premature stop codon at Q285 in exon 8 [15], while the other contains a splice site mutation in intron 10 resulting in the skipping of exon 10 (329–355 aa) [16]. Heterozygous mice of both genotypes exhibit a 50% reduction in *OPA1* transcript levels in the retinal tissue along with a similar reduction in OPA1 protein in a variety of other tissues. Interestingly, these models appear to recapitulate the slow visual degeneration but do not display loss of RGCs seen in human patients [17]. Despite these mouse models, questions still remain as to how depletion of the ubiquitously expressed OPA1 protein results in defects in mitochondrial function and why this depletion results in the apparent tissue specific phenotype. Furthermore, *OPA1* null mice and homozygotes lose viability at embryonic day (E) 9, which may explain the lack of identified patients with homozygous *OPA1* mutations [17]. The specific defects that occur before E9 resulting in mortality in these mice have not been characterized.

Zebrafish are an attractive model for studying vertebrate development and have been used for many years to describe developmental events that are difficult or impractical to view in mammalian models [18]. Zebrafish can produce hundreds of embryos in a single breeding and the embryos are transparent and develop outside of the mother, allowing for non-invasive observations of organ development [19]. *Opa1* is ubiquitously expressed in zebrafish [20] as a single-copy gene and is 78% identical and 87% similar to the most abundant human OPA1 isoform (OPA1-4) at the protein level. In order to investigate the role of Opa1 in embryonic development, we developed a zebrafish model of Opa1 depletion and determined the functional consequences of Opa1 mediated mitochondrial dysfunction.

## Results

### Changes in Opa1 protein during zebrafish embryonic development

**Figure 1:**
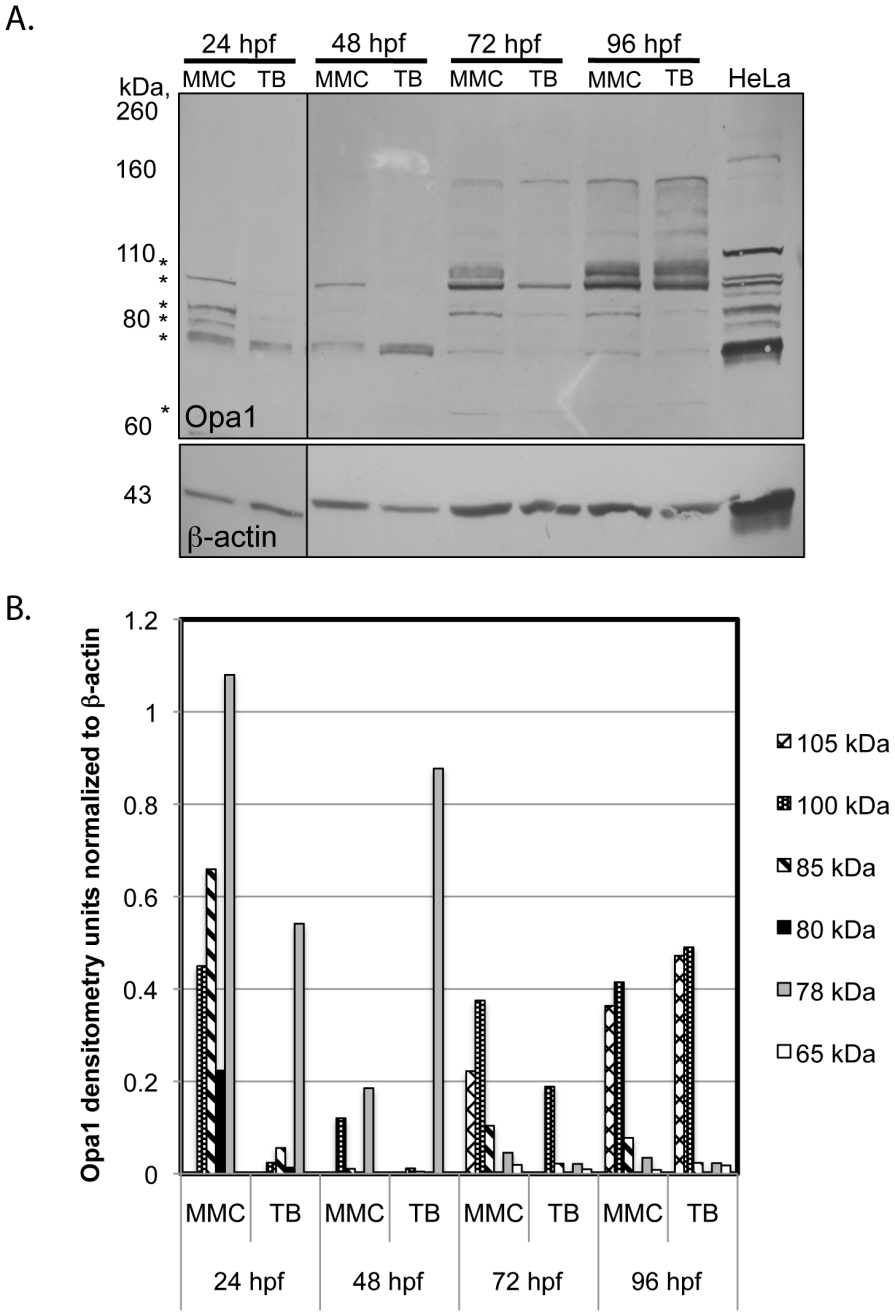
Western blot analysis. **A.** Representative Western blot for Opa1 yolk-less protein in MMC and Opa1 morphants. Opa1 protein is reduced at 24, 48, and 72 hpf. Differences were isoform specific. Samples from 24 hpf were imaged from a separate blot. Contrast was adjusted to improve visualization. *indicates isoforms selected for densitometry (see Figure 1B). Western blot results have been replicated with at least four independent injections **B.** Quantification of most intense Opa1 isoforms identified by Western blot. All values were first normalized to β-actin protein levels.

In order to examine the role of Opa1 in early embryonic development, a translation-blocking (TB) morpholino was microinjected into 1–4 cell stage zebrafish embryos to generate Opa1 morphants. A 5-bp mismatch control (MMC) morpholino was also microinjected at the same concentration into embryos from the same breeding pairs as a control for off-target effects. Specific knockdown of *Danio rerio* Opa1 was confirmed by Western blot analysis (Figure 1A). The high degree of conservation between human and zebrafish Opa1 allowed the use of a human OPA1 antibody to detect zebrafish Opa1. Yolk protein was found to interfere with the migration of the Opa1 protein, thus analysis was performed on embryos where the yolk cell had been removed prior to protein extraction. Like other vertebrates, *Danio rerio* Opa1 appears to exist as multiple isoforms (Figure 1A). Injection of the TB morpholino resulted in reduction of at least some of the Opa1 isoforms at all time points measured. At 24 hpf, the detectable isoforms of 100, 85, 80 kDa were all reduced to <10% of MMC levels (Figure 1B), whereas the doublet at approximately 78 kDa was only reduced to 50% of MMC levels. Interestingly, this 78 kDa doublet was four times more intense at 48 hpf compared to MMC, whereas the other isoforms were again reduced. At 72 hpf, all isoforms were reduced to 50% or less of MMC levels but by 96 hpf, only two isoforms remained lower than for MMC indicating the loss of morpholino effectiveness at this time point. This analysis also indicates that the larger isoforms increased in abundance over time regardless of morpholino injection (Figure 1A, B).

### Opa1 depletion results in defects in embryonic development

**Figure 2:**
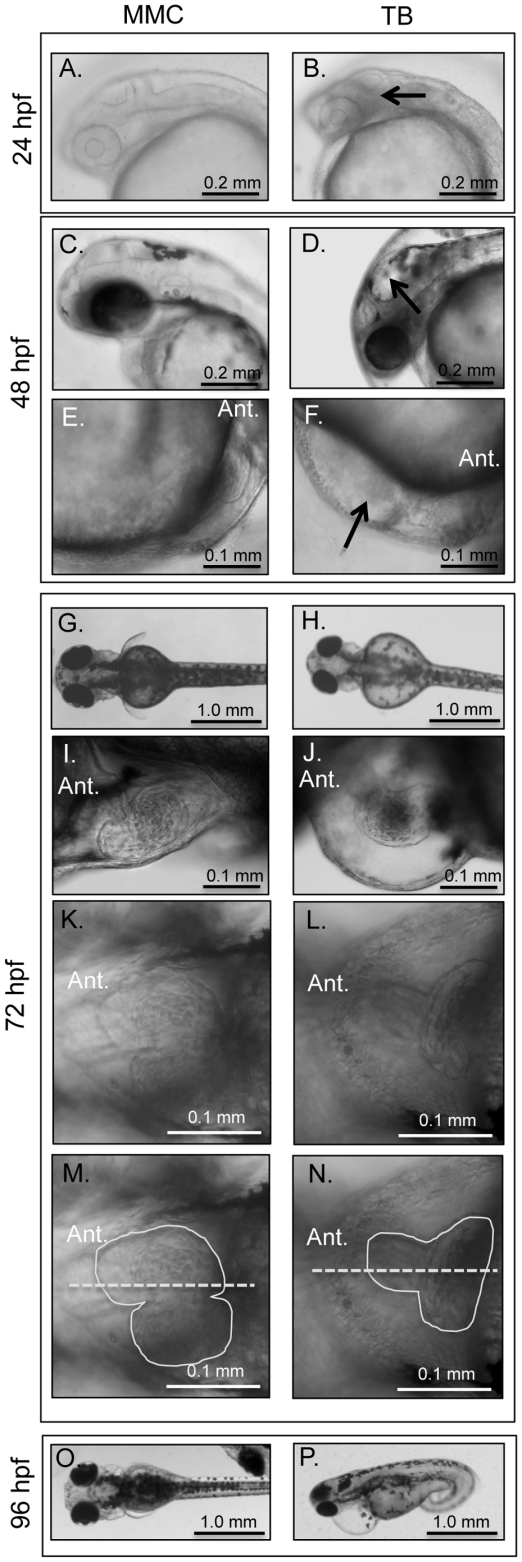
Phenotypic analyses of MMC (A, C, E, G, I, K, M, O) and TB (B, D, F, H, J, L, N, P) morphant embryos and larvae. Opa1 morphants at 24 hpf (**B**) have increased density or ‘graininess’ in the brain region (arrow) and smaller eyes. At 48 hpf, Opa1 morphants have hindbrain ventricle enlargements (arrow) and smaller eyes (**D**). Opa1 morphants at 48 hpf also have impaired circulation compared with MMC morphants and often has blood accumulation below the heart (arrow) (**F**). At 72 hpf, Opa1 morphants have larger yolk cells, smaller eyes, smaller hearts, small pectoral fin buds (**H**) and pericardial edema (**J**). Many Opa1 morphants had unlooped hearts (**L**). (**N**) is the same image as (**L**) with the heart margins outlined (solid line) and the midline indicated by a dashed line. By 96 hpf, the edema is still present and can involve the eyes (**P**).

**Figure 3:**
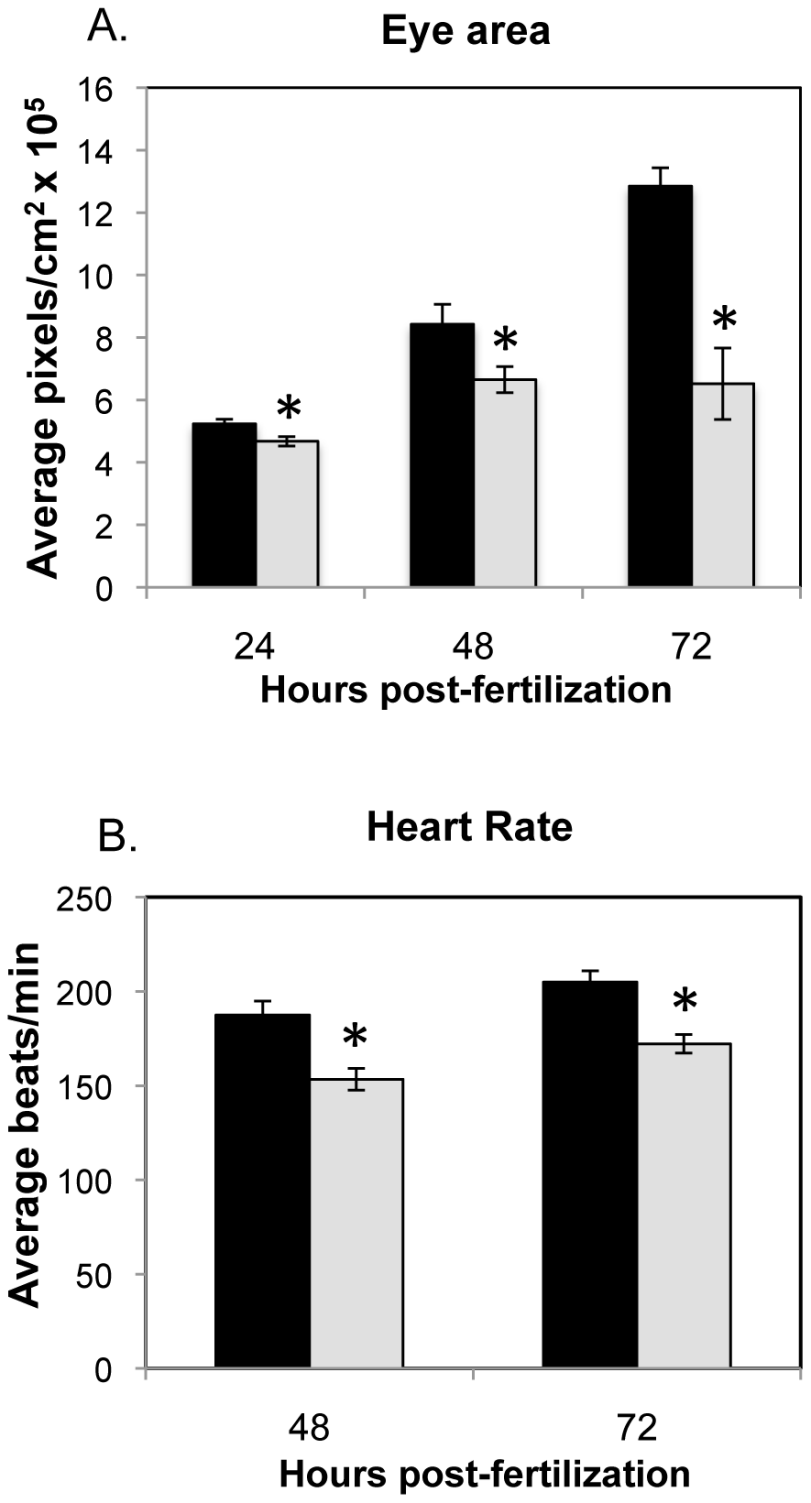
Eye area and heart rate analyses for MMC (black) and Opa1 morphants (grey). **A.** Eye area was measured by tracing the circumference of individual eyes using AxioVision software. N = 9–12. *p-value <0.05, **p-value <0.01 by Student's 2-tailed t-test. **B.** Heart rates were measured by counting beats per min for individuals injected with MMC or TB morpholino. N = 34 (48 hpf), n = 44 (72 hpf). *p-value <0.01 by Student's 2-tailed t-test.

Opa1 and MMC morphants were examined at various time points during development. At 12 hpf, no phenotypic or developmental differences were noted between the two groups. At 24 hpf, Opa1 morphants had less definition in and around the midbrain-hindbrain boundary and had significantly smaller eyes (Figure 2B, Figure 3A). Opa1 morphants were also significantly shorter (as measured by standard length) than MMC counterparts, which may indicate a developmental delay [21]. At 24 hpf, the zebrafish heart is an unlooped immature tube that has begun coordinated contractions. This contraction was noted in MMC fish but was less likely to be observed in Opa1 morphants at 24 hpf, possibly due to developmental delay. The brain abnormalities noted at 24 hpf were absent at 48 hpf, however the hindbrain ventricle was enlarged in Opa1 morphants (Figure 2D). Opa1 morphants at 48 hpf were still slightly delayed as indicated by shorter length, but also had significantly smaller eyes (Figure 2D), edema around the heart that often included erythrocyte accumulation in the area behind the pericardium (Figure 2F), and reduced cardiac contractility as measured by heart rate (Figure 3B). By 72 hpf, the hindbrain ventricle enlargement had subsided in Opa1 morphants but the eyes and head remained smaller than MMC morphants (Figure 2H). Opa1 morphant larvae were still slightly delayed in length although the difference was less significant than earlier time points (data not shown). The pericardial edema noted at 48 hpf was more extensive at 72 hpf in most cases (Figure 2J), and heart rates were still significantly reduced compared with MMC larvae (Figure 3B). Additionally, Opa1 morphants had larger yolk cells, suggesting reduced metabolism of yolk contents. Also noted were the smaller pectoral fin buds in Opa1 morphants (Figure 2H). Startle response, locomotor activity and swim duration were also reduced in Opa1 morphants; when a startle response was triggered, Opa1 morphants tended to swim erratically and often spun in circles briefly before stopping whereas MMC morphants had a robust response and tended to swim in a straight line until encountering the edge of the plate (Movies S1 and S2). Heart abnormalities such as smaller atrium and unlooped hearts were also noted in many Opa1 morphant larvae (Figure 2J, L, N). By 96 hpf, edema was noted in the eyes, pericardia, and yolk cell in Opa1 morphants (Figure 2P). These larvae did not survive past 7 dpf. A splice-blocking morpholino (SB) designed to span the junction between intron 12 and exon 13 was also injected at 4.2 ng, which produced morphants that exhibited the same phenotypes and behaviors as the morphants generated via TB morpholino (Figure S3). An mRNA rescue was attempted by co-injecting SB morpholino with full-length zebrafish *opa1* mRNA. mRNA dose was verified with RT-PCR. Interestingly, this co-injection resulted in increased death and a more severe phenotype compared to SB injected embryos (data not shown) rather than a rescue of the Opa1 phenotype.

As OPA1 deficiency in humans is largely associated with phenotypes involving the eye, histological analysis was conducted on the eyes of MMC and Opa1 morphants at 48 and 72 hpf. No degeneration of the optic nerve or loss of the RGC layer was observed in TB-injected larvae compared to MMC-injected larvae (Figure S4). The only eye phenotypes observed were smaller eyes overall (Figure 2H, Figure 3A) and edema around the eye at later stages.

### Altered mitochondrial morphology and gene expression in *opa1* morphants

**Figure 4:**
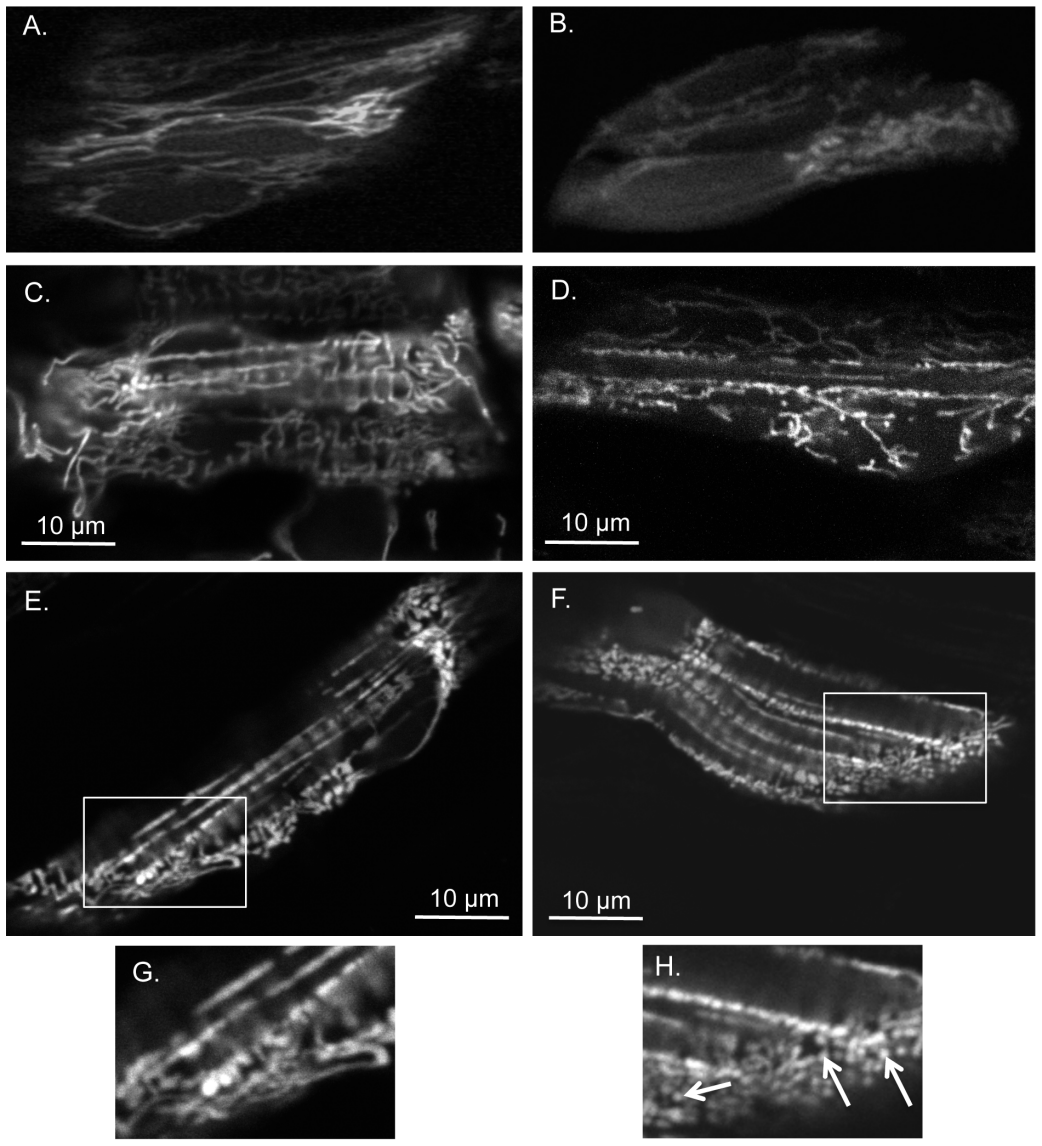
Opa1 morphants have more fragmented mitochondria and disorganized fibers when compared to MMC morphants. Cells from MMC morphants at 24 hpf (**A**), 48 hpf (**C**), 72 hpf (**E**) are compared to similar regions in Opa1 morphants at 24 hpf (**B**), 48 hpf (**D**), 72 hpf (**F**). (**A-B**) obtained with multiphoton confocal microscopy 400x with 3.8-4.0x zoom and (**C-F**) with single photon confocal microscopy 400x with 4.0x zoom. (**A-B**) cells within the eye, (**C-F**) skeletal myocytes. (**G**) and (**H**) are enlargements of boxed areas in (**E**) and (**F**) respectively. Note abnormal mitochondrial morphology in (**H**) as denoted by arrows.

In order to examine mitochondrial morphology, a mini-transposon containing the mitochondrial localization signal for zebrafish CoxVIII fused with EGFP was co-injected with the TB or MMC morpholino resulting in EGFP targeted to the mitochondrial matrix [22]. Muscle cells from MMC and Opa1 morphant embryos and larvae were imaged by confocal and standard fluorescence microscopy at 24, 48 and 72 hpf. In general, mitochondria from Opa1 morphants appeared to have less overall fluorescence than their MMC counterparts at all time points examined. Morphological differences between mitochondria from Opa1 and MMC morphants were noted; one of the differences we observed was increased mitochondrial fragmentation as evidenced by the small circular puncta in muscle cells of Opa1 morphant fish (Figure 4).

**Figure 5:**
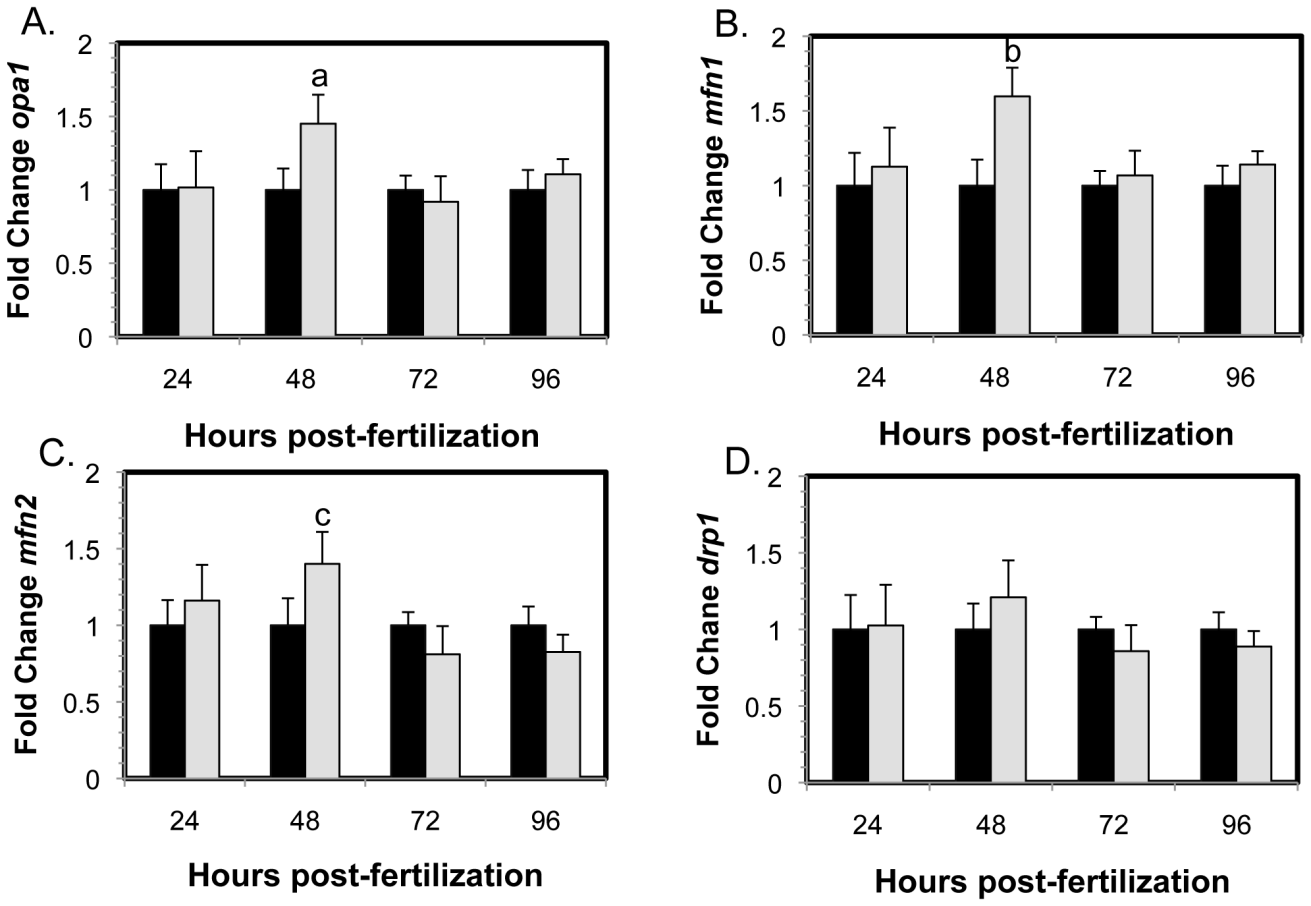
Gene expression changes of mitochondrial morphology genes in MMC morphants (black) and Opa1 morphants (grey) normalized to MMC morphant levels. Significant increases in gene expression of mitochondrial fusion proteins (**A**) *opa1* (a, p = 0.003), (**B**) *mfn1* (b, p = 0.001) and (**C**) *mfn2* (c, p = 0.01) were observed in Opa1 morphants compared to MMC morphants. No differences were observed for (**D**) *drp1*, a mitochondrial fission protein. Error bars are shown +/− SEM, n = 5. P-values obtained by ANOVA.

Gene expression analyses indicated that expression of genes involved in mitochondrial fusion (*opa1*, *mfn1* and *mfn2*) were upregulated at 48 hpf (Figure 5A–C), which may explain the increased levels of one of the smaller Opa1 isoforms at 48 hpf (Figure 1A–B). However, gene expression of *drp1*, which is involved in mitochondrial fission, did not change over 96 hr (Figure 3D), revealing an imbalance in mitochondrial dynamics with a shift toward mitochondrial fusion over fission, at 48 hpf. This appears to result in an increase in at least one of the smaller Opa1 protein isoforms (Figure 1).

### Genes involved in mitochondrial biogenesis are upregulated in response to Opa1 depletion while mtDNA replication and transcription are largely unaffected

**Figure 6:**
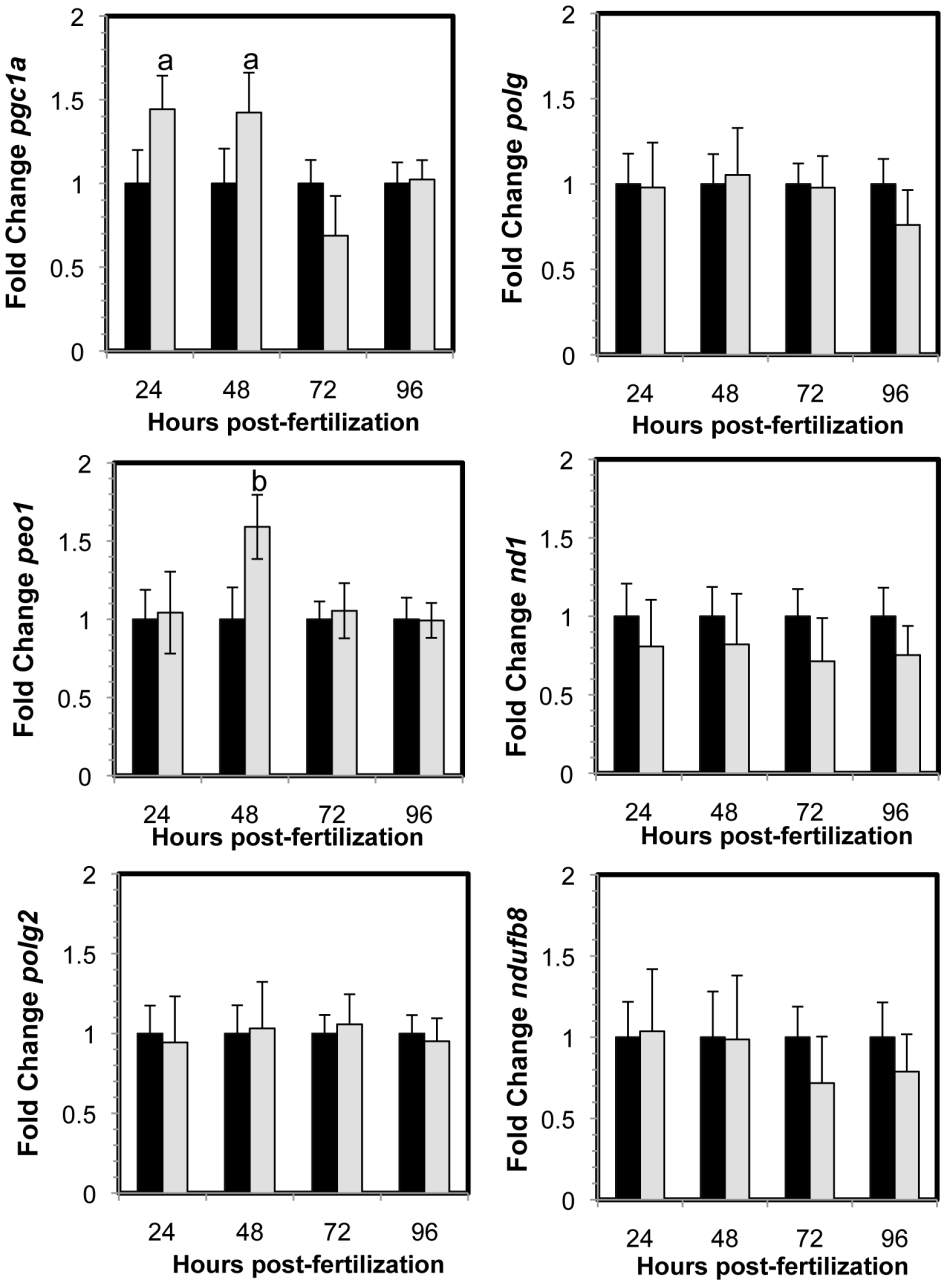
Gene expression changes in MMC morphants (black) and Opa1 morphants (grey) normalized to MMC morphant levels. Significant increases in gene expression *pgc1a* (a, p = 0.02) and *peo1* (b, p = 0.002) were observed in *opa1* morphants compared to MMC morphants. Error bars are shown +/- SEM, n = 5. P-values obtained by ANOVA.

Peroxisome proliferator-activated receptor gamma coactivator 1-alpha is encoded by *pgc1a*, a key regulator of mitochondrial biogenesis. Gene expression analysis shows that *pgc1a* was upregulated at 24 and 48 hpf (Figure 6). Further analysis of genes involved in mtDNA replication revealed that expression of the gene encoding the mtDNA helicase, *peo1*, was upregulated only at 48 hpf (Figure 6). However, gene expression of *polg* and *polg2*, which encode the mtDNA polymerase catalytic and accessory subunits respectively, did not change over time. Expression of *nd1*, an mtDNA encoded gene, also did not change. This is in agreement with the mtDNA content and deletion assays, which did not reveal any changes in the copy number or integrity of mtDNA (Figures S1 and S2). There was also no change in gene expression of *ndufb8*, a nuclear gene encoding a mitochondrial protein (Figure 6). Interestingly, at 48 hpf when *peo1* was upregulated (Figure 6), transcription of the mitochondrial fusion genes *mfn1*, *mfn2* and *opa1* were also upregulated (Figure 5).

### Opa1 morphants show bioenergetic defects without impairment to mitochondrial efficiency

**Figure 7:**
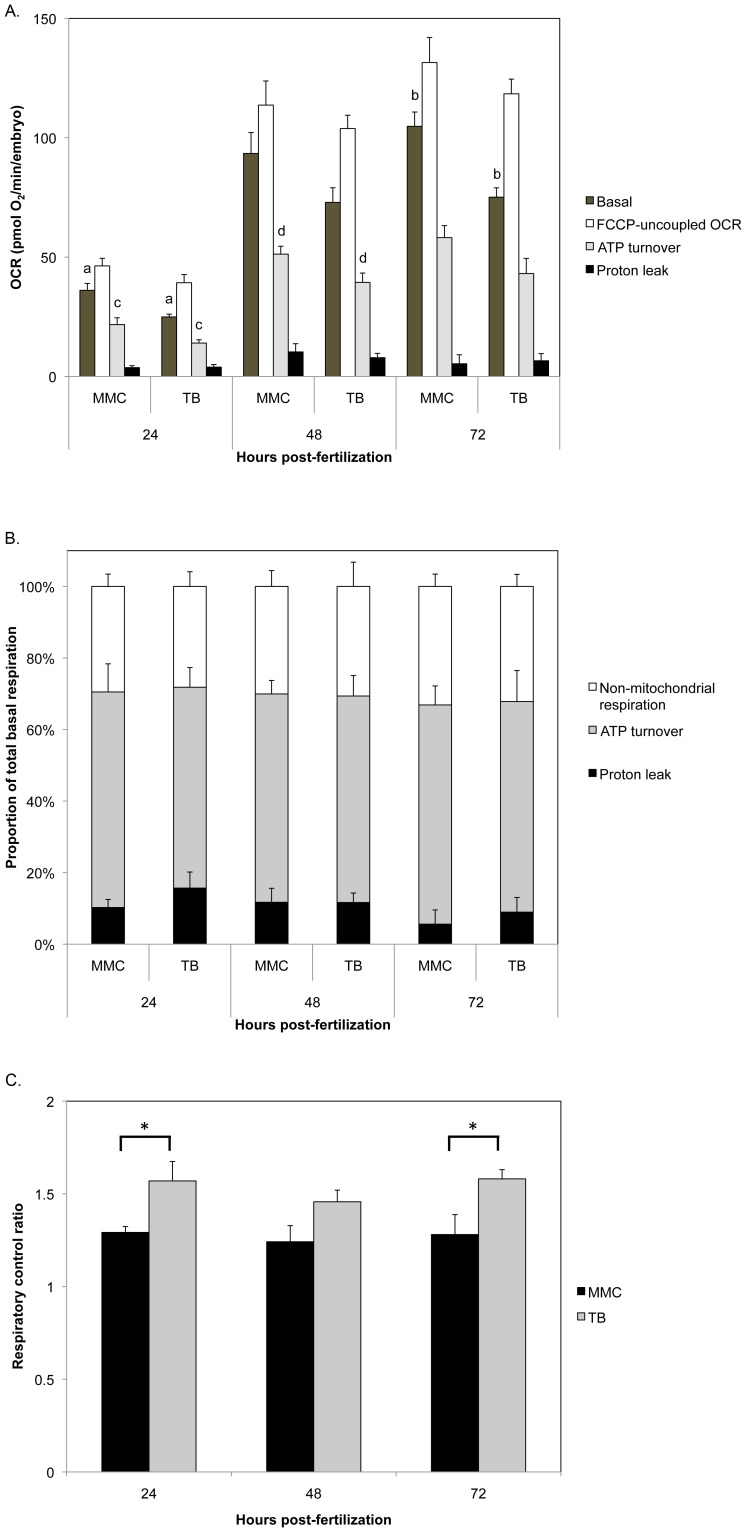
Bioenergetic analysis of Opa1 morphants. Oxygen consumption rates (OCR) were measured in 24, 48, and 72 hpf morphants (mean +/− SEM, n = 5–10). **A.** Opa1 morphant basal respiration was significantly decreased compared to MMC morphants at 24 and 72 hpf (p = 0.004 (a) and p = 0.0007 (b), respectively). Maximal respiratory capacity and proton leak were not different between groups at any time point. ATP turnover was significantly decreased for Opa1 morphants at 24 and 48 hpf (p = 0.03 (c) and p = 0.04 (d), respectively). **B.** The proportion of total basal respiration due to non-mitochondrial respiration, ATP turnover and proton leak were similar between Opa1 and MMC morphants. **C.** Respiratory control ratio was significantly greater for Opa1 morphants than MMC at 24 and 72 hpf (asterisks, p<0.03). P values were determined by Student's 2-tailed t-test.

Respiration, expressed as oxygen consumption rates (OCR), was measured for embryos at 24, 48, and 72 hpf using our recently published method [23]. Total basal respiration was significantly decreased in Opa1 morphants when compared to MMC morphants at 24 and 72 hpf (Figure 7A, ‘Basal respiration’). Oligomycin inhibits ADP phosphorylation at the mitochondrial ATP synthase; by treating embryos with this inhibitor we can determine the amount of basal OCR that can be attributed to ATP turnover (that is, ATP synthesis and subsequent ATP utilization) and the remaining OCR is mitochondrial leak (having subtracted non-mitochondrial OCR) [23]. ATP turnover was significantly lower in Opa1 morphants at 24 and 48 hpf compared to MMC morphants (Figure 7A, ‘ATP turnover’). Proton leak was not different between Opa1 and MMC morphants (Figure 7A, ‘Proton leak’). Interestingly, if we plot the proportion of basal respiration due to non-mitochondrial and mitochondrial OCR (ATP turnover and proton leak comprise mitochondrial OCR), we saw that the proportions were similar between the two groups (Figure 7B). Maximal mitochondrial respiratory capacity was not significantly different between the two groups at any time point examined (Figure 7A, ‘FCCP uncoupled OCR’). Respiratory control ratio (RCR), which is the ratio of FCCP-uncoupled OCR to basal OCR and is a measure of mitochondrial efficiency, was significantly higher in Opa1 morphants compared to MMC at 24 and 72 hpf (p<0.03, Figure 7C). However, RCR was not different between 24, 48 and 72 hpf for Opa1 morphants, nor for MMC morphants.

## Discussion

Heterozygous *OPA1* mouse models and work in *Drosophila* and *C. elegans* describe the relationship between OPA1 and mitochondrial dynamics and help to explain how dysfunctional mitochondria might affect the organism as a whole [17], [24]–[26]. However, questions still remain regarding the connection between mitochondrial dynamics and the physical phenotypes that are correlated with OPA1 depletion in humans. A number of studies have attempted to examine the roles of the various OPA1 isoforms in cell culture and mouse models. Different isoforms can be generated by alternative splicing or by post-translational cleavage or modification, and each isoform varies in abundance in separate tissues strongly suggesting the importance of specific isoforms in particular tissues [4], [27], [28]. Additionally, it appears that mitochondrial membrane potential may affect OPA1 processing suggesting a connection between the functional state of the mitochondria and mitochondrial morphology as regulated by variations in the presence of longer OPA1 isoforms [1], [29], [30].

In this study, we decreased Opa1 protein expression in zebrafish embryos using a morpholino designed to block translation of zebrafish Opa1 while not affecting the splicing of the mRNA or post-translational cleavage or modification. We demonstrated by Western blot significant alteration of Opa1 isoform abundance between 24–96 hpf in embryos injected with the Opa1 morpholino. Each isoform was more abundant at different stages within the developmental window monitored, highlighting the importance of the different isoforms in embryogenesis. Interestingly, at 48 hpf the 78 kDa isoform, which for 24 hpf Opa1 morphants was reduced to 50% of MMC levels, appeared four times as abundant compared to the MMC lane. This is despite the other detectable isoforms being reduced to 43% or 10%. The data from gene expression analysis indicated an approximately 50% up-regulation of genes involved in regulating mitochondrial biogenesis (including *opa1*) at this 48 hpf time point (Figure 5). It is possible that this increased expression, perhaps in response to the decrease in Opa1 at 24 hpf, results in *de novo* transcription of *opa1*. It is not clear why this potential attempt to compensate is not maintained past 48 hpf; it is possible that this response might only be a transient stop-gap that cannot continue indefinitely. At 72 hpf, all isoforms are reduced in abundance and although the two larger isoforms recover to MMC levels at 96 hpf, the smaller isoforms remain below MMC levels.

The appearance of particular Opa1 isoforms may be critical at specific times in embryonic development. This hypothesis is supported by the observation that co-injecting full-length *opa1* mRNA in combination with the splice blocking (SB) morpholino resulted in increased mortality and a more severe phenotype rather than a rescue. Chen et al. also observed a detrimental effect of overexpression of full-length Opa1 in H9C2, a rat embryonic heart cell line; after induction of ischemia, overexpression of OPA1 induced rather than protected against apoptosis [31]. We suggest that the more severe phenotype observed in Opa1 morphants co-injected with full-length cDNA may be due to the fact that the full-length *opa1* cDNA does not contain introns and thus alternatively spliced forms of Opa1 would not be derived from this construct. In addition to not allowing for proper splicing, overall timing of *opa1* expression may also account for the increased mortality in co-injected embryos; as shown in Figure 1, different Opa1 isoforms are needed at precise times in embryonic development. We have observed that zygotic *opa1* expression begins between 12 and 24 hpf. By injecting the full-length *opa1* transcript before the four cell stage we may in effect cause mis-timed overexpression of *opa1* transcript, which may result in induction of apoptosis as shown by Chen et al. [31].

### Developmental consequences of Opa1 depletion may be due to bioenergetic defects

We observed a small delay in development with Opa1 morphants compared to MMC morphants from the same clutch, although the delay appeared to become less pronounced at later time points (72 and 96 hpf). Prior to 24 hpf, somite number is a straight forward way to estimate developmental stage [21]. However, Opa1 morphant fish did not exhibit developmental delay until time points after 24 hpf at which point somite number becomes an unreliable measure of development and embryo/larval length should be used [21], [32]. Interestingly, studies by Mendelsohn et al. (2008) demonstrated that treatment of zebrafish embryos with chemical inhibitors of oxidative phosphorylation, and by extension ATP synthesis, resulted in developmental arrest [33]. They also hypothesized that mitochondrial energy production acts a common signal for oxygen levels in the developing embryo. Viewed in this light, our observation that Opa1 morphant embryos are developmentally delayed aligns with our data indicating a defect in energy metabolism in these embryos. Previous research by Chen et al. showed that siRNA knockdown of OPA1 in mouse embryonic fibroblasts led to decreased basal respiration [6]. Similarly, in our zebrafish Opa1 morphants, basal respiration was reduced at 24, 48, and 72 hpf (Figure 7A). As Opa1 morphants had movement defects (Movie S2) such as akinesia (inability to initiate movement) and Bradykinesia (slow movement), this decrease in basal respiration is most likely due to decreased movement and ATP demand. The reduction in basal respiration was not due to differences in proton leak (Figure 7A and B). We did not observe a change in maximal mitochondrial respiratory capacity in Opa1 morphants (Figure 7A), which is in contrast to the mouse embryonic fibroblast study, where a decrease in maximal respiration was observed when OPA1 was depleted in these cells [6]. This difference may be due to the fact that we have generated a whole organism deficient in Opa1 in contrast to a monolayer of fibroblasts cultured in enriched media. In the whole organism (or whole embryo), there may be compensatory mechanisms not available to cells in culture that allow for a decrease in mitochondrial respiration without affecting maximal respiratory capacity. Furthermore, we observed a significant increase in RCR for Opa1 morphants compared to controls at 24 and 72 hpf (Figure 7C); this aligns with our data that shows a decrease in basal respiration due to a decrease in ATP turnover/demand. Chevrollier et al. showed that the RCR of ADOA OPA1 mutant fibroblasts were significantly lower than for control fibroblasts [34]. Again, this may be due to differences in working with a single cell type growing in culture versus whole organisms/embryos.

We observed that larval length became less divergent between Opa1 and MMC morphants at the later time points. Developmental delay is a hallmark of mitochondrial disease [35] and can lead to functional deficiencies due to the specific timing of events in embryogenesis [36]. In Opa1 morphants, metrics such as eye size and heart rate did not reach MMC levels at any time point (Figure 3). Additionally, we visually observed slower circulation and pooling of red blood cells in Opa1 morphant larvae, a possible consequence of reduced heart rate or other unidentified vascular defects. The edema observed in the pericardium and yolk, as well as the hindbrain ventricular enlargement at 48 hpf, could be attributed to these circulation defects, which are consistent with other zebrafish lines bearing heart defects [37]. Under-utilization of the yolk also aligns with our other data that suggests that Opa1 depletion alters energy metabolism. We also observed that Opa1 morphant fish had decreased locomotor activity. Interestingly, akinesia and Bradykinesia are also observed in Parkinson's disease, which can also be caused by mitochondrial defects [38], [39].

As human patients with OPA1 mutations develop optic atrophy, we took a closer look at eye development. Opa1 morphants had smaller eyes and eye edema at later stages. However, in contrast to human patients with mostly mono-allelic OPA1 mutations, Opa1 morphant zebrafish did not show any observable RGC or optic nerve defects by histology at any time point. Humans with OPA1 mutations develop optic atrophy typically after age 10 and loss of the RGC layer is not observed until postmortem examination [17]. Since our experiments make use of morpholinos, we are limited to observing Opa1 depletion in zebrafish only during a brief window of early development (4–5 days). Such changes to the optic nerve and RGC layer appear to occur later in life and our current model does not survive long enough for these defects to be observed.

### Opa1 depletion results in altered mitochondrial morphology

By using a mitochondrial matrix-targeted EGFP construct, we showed that depletion of Opa1 in the zebrafish embryo resulted in generally diffuse appearance of the mitochondria with the majority appearing fragmented (Figure 4). This is in agreement with studies in cell culture and *C. elegans* models of OPA1 depletion [6], [25]. Long mitochondrial networks are important for improved mitochondrial function and resistance to stress induced by protein synthesis inhibition. OPA1 along with MFN1 have been shown to be critical components of this stress response [40]. Therefore, Opa1 morphants with fragmented mitochondria may be experiencing the consequences of reduced mitochondrial output.

### Opa1 depletion affects expression of genes important in regulating mitochondrial dynamics and biogenesis

Mitochondria are constantly being turned over to maintain a population of highly efficient organelles [41]. The processes of fission and fusion can help to mix mitochondrial contents and remove portions of organelles that are functioning poorly [41], [42]. Opa1 plays a key role in regulating this process. By impacting zebrafish Opa1 protein levels, we expected to initiate a response in other genes important in fusion and mitochondrial biogenesis, as is the case in cardiomyopathy, where mitochondria proliferate in response to mitochondrial dysfunction [43].

*Pgc1a* is a major regulator of mitochondrial biogenesis [44]. Gene expression of *pgc1a* was upregulated at 24 and 48 hpf (Figure 6) and we observed increased gene expression of *peo1*, *mfn1*, *mfn2* and *opa1* at 48 hpf (Figure 5), most likely in response to increased *pgc1a* and suggesting increased mitochondrial biogenesis. However, we did not observe a corresponding increase in mtDNA content or an increase in gene expression of the mtDNA polymerase catalytic and accessory subunit (*polg* and *polg2*; Figure 6). Consistent with this, we also did not see an increase in the gene expression of the mtDNA-encoded gene *nd1* or any changes in expression of *ndufb8*, a nuclear-encoded mitochondrial protein (Figure 6). It is known from cell culture studies that Opa1 depletion leads to fragmented mitochondria where some mitochondria do not contain mtDNA [45]. There may be sufficient mtDNA per cell, but not per mitochondrion, which suggests that the cell could regulate the amount of mtDNA but not the amount of mtDNA per mitochondrion. However, we did observe an increase in expression of *peo1*, encoding the mtDNA helicase. Peo1 is essential for up-regulating mtDNA replication and has been shown to be activated by Pgc1a via an interaction with Nrf2 [46]–[48]. It is also possible that mitochondrial biogenesis was not sustained long enough to significantly stimulate mtDNA replication beyond control levels.

MtDNA deletions were not observed in Opa1 morphant zebrafish. Additional experiments were performed using DNA extracted from the heads or hearts of Opa1 and MMC morphants. Again, no deletions were detected in these tissues. In other model systems, mtDNA deletions are often found in older aged organisms or not until young adulthood in patients with mitochondrial disease [14]. Therefore we may not detect mtDNA deletions in the early stages of embryogenesis.

With this novel zebrafish model, we have identified a connection between Opa1 depletion, mitochondrial dysfunction, and ultimately development, which may explain the observed embryonic lethality in mice and presumed embryonic lethality in humans. This study places emphasis on mitochondrial function during key developmental stages and we are able to address previously unknown connections between Opa1-dependent mitochondrial and developmental functions. We believe that further studies using this new model will not only point to the vital nature of mitochondrial function during development but also lead to a more complete understanding of the consequences of OPA1 mutations or variants in humans.

## Experimental Procedures

### Zebrafish maintenance and care

Wild-type zebrafish (AB strain) were obtained from the Zebrafish International Resource Center, which is supported by grant P40 RR012546 from the NIH-NCRR. Zebrafish were maintained and crossed according to standard methods [49]. Fertilized eggs were collected and placed in E3 embryo medium, and maintained in an incubator set at 28.5 °C with a 14/10 hr light/dark cycle. Embryos were staged using the criteria of Kimmel et al. [21]. All animal studies were approved by the Medical University of South Carolina Institutional Animal Care and Use Committee (AR #2850) and performed in accordance with the guidelines.

### Morpholino injections

Opa1 translation blocking (TB), 5-bp mismatch control (MMC) and splice-blocking (SB) morpholinos were designed with Gene Tools, LLC (Table 1). Morpholinos were diluted in sterile dH_2_O to a stock concentration of 6 mM and further diluted to 1 mM. Working solutions of phenol red were prepared by diluting neat phenol red (Sigma) 1:10 with 1x sterile Danieau solution (58 mM NaCl, 0.7 mM KCl, 0.4 mM MgSO_4_, 0.6 mM Ca(NO_3_)_2_, 5 mM HEPES, pH 7.2). Morpholinos were titrated to determine the amount of morpholino required for phenotypic differences without toxicity. We tested 4.2, 8.5, 10.6, 11, 12.8, 17 ng for TB; 3.2, 4,2, 8.5 ng for SB; and 4.2, 8.5, 10.6, 11, 12.8, 17 ng for MMC. 12.8 ng and 17 ng MMC morphants displayed high mortality and appeared deformed, thus 8.5 ng final concentration of TB and MMC morpholino were used for injection, and 4.2 ng for SB morpholino. 3 nL TB, MMC or SB morpholino was injected into the 1–4 cell stage embryo (1–2 hpf). Final concentration of morpholino in injection solution was 0.33 mM for TB or 0.17 mM for SB by dilution with phenol red.

**Table 1:** Antisense morpholino sequences.

Morpholino	Sequence
Opa1 translation blocking (TB)	GATGAGTTTAGGATCTCTTTGCAGT
TB 5 bp mismatch control (MMC)	GATCACTTTACGATCTGTTTCCAGT
Opa1 splice blocking intron 12/exon 13 (SB)	ACAGTCTGCGAAAAGAAGTCAATCA

### mRNA rescue

A zebrafish full length clone for *opa1* was obtained from OpenBiosystems (Clone ID 7047256) in the pExpress-1 vector. The clone was subcloned and sequenced to verify the correct insert and orientation. Once confirmed, the clone was linearized by digestion with *Xho*I and purified. The Ambion mMessage mMachine kit (Invitrogen) was used to transcribe mRNA using the SP6 promoter. mRNA quality and quantity was measured by nanodrop. mRNA was co-injected with the SB morpholino 4.2 ng at 25 and 35 pg per embryo keeping the 3 nL injection volume. To confirm injected mRNA expression, embryos were collected, cDNA made and PCR performed using primers (F1: GGTCAGACAGTCAGCCCTGAGACC; R1: GGTCCTTTTGCCCTGAGGGTCC) which span the intron/exon boundary targeted by the morpholino.

### Heart rate measurements

Heart rates were measured by counting the number of atrial contractions in 30 seconds in embryos or larvae under 100x magnification using a Zeiss Axio Observer A.1 inverted microscope. Beats per min were calculated and mean values plotted +/− standard error of the mean (SEM).

### Measurements of eye size

Zebrafish eye size was determined using Axiovision software (Zeiss). Individual images of embryos or larvae were examined and a free-hand circle drawn around the circumference of each eye. The program calculated the area of each circle and the mean values were plotted +/−SEM.

### Behavioral assay

We monitored startle response in MMC and TB morphant larvae at 72 hpf. One 72 hpf larvae was placed into each well of a 6 well plate containing 5 mL E3 embryo media. The plate was then placed in a Daniovision locomotion tracking instrument equipped with a FireWire infrared camera (Noldus Information Technology). Video was recorded at 60 frames/second using MediaRecorder software (Noldus Information Technology). Stimulus was administered by touching the larvae with a short piece of plastic bent to fit inside the wells while not disturbing the plate itself inside the Daniovision system.

### Protein extraction

Embryos were de-yolked by positioning a pipet tip and pipeting free the yolk cell. 20 embryos were pooled for each time point and quickly frozen and stored at −80 °C. Protein lysates were prepared by homogenization in 35 µL RIPA buffer (150 mM NaCl, 1 mM EDTA, 50 mM Tris HCl pH 7.5, 1% NP-40) supplemented with 1:100 protease inhibitor cocktail (Sigma P8340) using microfuge pestles and a hand-held motorized homogenizer. Samples were centrifuged at 4 °C for 10 min and the supernatant removed to a new tube. Concentrations were determined using the BCA assay (Sigma) on a 1:10 dilution of each sample.

### Western blotting

15 µg of embryo lysate was loaded per lane on 12% MiniPROTEAN TGX gels (BioRad). Electrophoresis was carried out in a BioRad unit at 166 V for 90 min. The gel was soaked in 2x transfer buffer (Invitrogen), 10% methanol, 1x antioxidant (Invitrogen) for 15 min. Transfer of protein from the gel to PVDF membrane was performed using an iBlot apparatus (Invitrogen), program P3 for 7 min. The blot was then removed and rinsed in TTBS (10 mM Tris-HCl pH 8.0, 137 mM NaCl, 0.05% Tween-20) for 5 minutes before blocking in 5% nonfat dry milk/TTBS for 1 hr at room temperature. Blots were incubated overnight at 4 °C in β-actin antibody (Sigma A2228) at a 1:1,000 dilution or Opa1 antibody (Novus Biologicals) at 1:400. The β-actin blot was incubated in 1:500 anti-mouse HRP secondary antibody (Thermo Fisher) and the Opa1 blot was incubated in 1:1,500 anti-rabbit HRP secondary antibody (Invitrogen) at room temperature for 1 hr. Chromogenic HRP substrate (Invitrogen WP20004) was added to blots of visualize immunoreactive bands. Blots were scanned and bands were quantified using ImageJ software (NIH). Opa1 densitometry values were normalized to β-actin (loading control) values.

### DNA extraction

Whole embryos (10 per time point), heads (20 per time point), or hearts (20 per time point) from euthanized larvae were collected at 24, 48, 72, 96 hpf and were frozen and stored immediately at −80 °C until processing. Samples were incubated overnight at 55 °C in 250 µL digestion solution (50 mM Tris-HCl pH 8.0, 200 mM NaCl, 100 mM EDTA pH 8.0, 1% SDS, 0.2% DTT, 2 mg/mL proteinase K (EMD chemicals)). DNA was prepared using standard phenol chloroform extraction [50] and quantified by PicoGreen assay (Invitrogen).

### Mitochondrial imaging

The mitochondrial EGFP expression vector (a generous gift of Dr. Dale Hailey, University of Washington) was transfected into Top10 cells (Invitrogen). Subclones were screened for correct plasmid size [22]. Plasmid DNA was extracted from confirmed clones using Qiagen Miniprep kit. The plasmid DNA was diluted to 125 ng/µL with dH_2_O and stored in aliquots at −20 °C until use. Into 1–4 cell stage embryos, 25 pg purified plasmid DNA was co-injected with either MMC or TB morpholino in a 3 nL injection volume. Embryos and larvae were viewed using a Zeiss LSM 510 NLO Laser Scanning Confocal Microscope with multiphoton excitation at 900 nm or single photon excitation at 488 nm.

### Analysis of relative mtDNA copy number

Genomic DNA was diluted in water and 8.5 ng was used in a QPCR reaction using SsoAdvanced SYBR Green Supermix (BioRad) with appropriate primers (300 mM each) with the BioRad CFX96 RealTime instrument (Table 2). The following cycle conditions were used: 95 °C/3 min, 30 cycles of 95 °C/15 s, 62 °C/30 s, followed by 95 °C 30 s and a dissociation curve. Samples were run in duplicate and averaged for each run individually. MtDNA content was obtained using the 2^−ΔΔCt^ method [51] whereby all ND1 (mtDNA target) C_t_ values were first normalized to EF1a (nuclear DNA target). TB-injected samples were then normalized to MMC-injected samples. Data from multiple experiments were collated and SEM calculated using the procedure described in Bookout and Mangelsdorf [52]. Primer pairs were verified and 95–105% efficiency confirmed by the standard curve method.

**Table 2:** Primers for RT-PCR.

Gene	Forward primer	Reverse primer
*nd1*	GGGCACCCATACCCATGCCCTAT	TGCGCTACAGCTCGTAAGGC
*ef1a*	AAGCCGCTGAGGTAAGCGTTCAAC	TTGAGCCGACAAACGCGTGCTG
*pgc1a*	GGCCCAGCGAGCCAAACCAA	TGGCTTTGTGAGGAGGCGTGG
*mfn1*	CTGGGTCCCGTCAACGCCAA	ACTGAACCACCGCTGGGGCT
*mfn2*	GCTGGGACGCATCGGCCAAT	GAGCGATCCACCACCCGCAG
*opa1*	GCCGGAAGTGTAGTTACCTG	AGGTGGTCTCTGTGGGTTGT
*drp1*	AGCCAGTCAGGTGATCGCCGA	CGCAGGGTTCGCGTGAAGGG
*peo1*	CCTTGGTTTGGCGGACGGGA	AACCTCGGCGTCCTTGCGTC
*polg*	GGAAACCAGGCGGCGTCATGT	ACGAACGTCTGCCACCGGTGA
*polg2*	GTGGAGGAAGTTTGCTTTAGGCCCG	GGGTCCACAGTGTCTCCAGCGT
*l13b*	AAGCAAGTGCTGTTGGGCCACA	TGATCCACGGGAAGGGTTGGTGT
*ndufb8*	TGCCCGGTCCGTACCCCAAA	AATCGCCACTCCCCCAGCCA

### MtDNA deletion assay

MtDNA deletions were assayed using an XL-PCR assay [53], [54]. Briefly, 17.5 ng of genomic DNA was used in a reaction with 0.2 mM dNTPs, 0.5 mM primers (Table 3) and Phusion Hot Start II High-Fidelity DNA polymerase (Finnzymes) with buffer HF. Two separate reactions were performed for each sample using either the 7.6 kb or the 10.3 kb primer set. PCR cycling was performed in a BioRad C1000 thermal cycler using the following conditions: for 7.6 kb primers, 98 °C/30 seconds, 30 cycles of 98 °C/10 s, 72 °C/3 min 45 s, followed by a final extension of 72 °C/10 min; for 10.3 kb primers, 98 °C/30 s, 30 cycles of 98 °C/10 s, 72 °C/5 min 15 s, followed by a final extension of 72 °C/10 min. PCR products were analyzed by separation on 0.8% agarose gels.

**Table 3:** Primers for XL-PCR for mtDNA deletion assay.

MtDNA fragment	Forward primer	Reverse primer
7.6 kb	TGGTGCTTACCGACCACCCT	GCGCTAGGGAGGGGTCTAACCT
10.3 kb	TTAAAGCCCCGAATCCAGGTGAGC	GAGATGTTCTCGGGTGTGGGATGG

### RNA extraction and gene expression analysis

RNA was extracted from 20 whole embryos for all time points using Trizol (Invitrogen) followed by RNeasy Mini Kit (Qiagen). Briefly, frozen embryos were homogenized in 800 µL Trizol using in-tube pestles and a motorized homogenizer. Following a 5-min incubation at room temperature, 200 µL chloroform was added and the samples separated by centrifugation at 12,000 g for 10 min. The aqueous phase was transferred to a new tube and 250 µL 100% ethanol was added and the samples mixed. This mixture was then transferred to the Qiagen minicolumn assembly and the protocol followed as described with the kit. Samples were eluted in 30 µL RNase free water and concentration was determined using the Nanodrop instrument (Thermo Fisher). cDNA was prepared using the RETROscript kit (Ambion) with 500 ng total RNA. cDNA was then diluted 1:2 with sterile dH_2_O and 2.5 µL of this cDNA was used in the QPCR reaction described under ‘Analysis of relative mtDNA copy number'. The 2^−ΔΔCt^ method was used, whereby all gene C_t_ values were first normalized to C_t_ values of *l13b* (for 24 hpf) or the geometric mean of the C_t_ of *l13b* and *ef1a* (for 48–96 hpf) [51]. TB-injected samples were then normalized to MMC-injected samples. Data from multiple experiments were collated and SEM calculated using the procedure described in Bookout and Mangelsdorf [52].

### Respirometry

Oxygen consumption rate measurements on 24, 48 and 72 hpf zebrafish embryos were performed on the XF-24 Extracellular Flux Analyzer (Seahorse Bioscience) using standard methods [23].

### Statistical analyses

Statistical analyses were performed using Kaleidagraph v4.0 (Synergy Software) or Excel (Microsoft), either one-way ANOVA followed by Student-Newman-Keuls post hoc test for multiple comparisons or Student's t-test for single comparisons. Differences were considered statistically significant when p<0.05.

## Supporting Information

Figure S1 **MtDNA content analysis in MMC morphants (black) and Opa1 morphants (grey).** Whole fish, heads or hearts only were pooled from 10, 20 or 20 larvae respectively and DNA analyzed by QPCR for mtDNA:nuclear DNA ratio. Multiple independent injection experiments were performed for each data set. Fold change was calculated using the 2^∧ΔΔCt^ method comparing Ct values obtained from *nd1* (mtDNA-encoded gene) and *ef1a* (nuclear single copy gene). Error bars show SEM. N = 5 for whole fish, n = 6 for heads, n = 3 for hearts. No significant differences were obtained.
(TIF)

Figure S2 **MtDNA deletion analysis.** DNA was extracted from whole fish, heads or hearts at various time points (12, 24, 48, 72 and 96 hpf) and analyzed by XL-PCR using two sets of primers, one amplifying a 7.6 kb region of mtDNA and the other a 10.3 kb region of mtDNA. PCR products were analyzed by electrophoresis on 0.8% agarose gels. Representative gels are shown from whole fish samples. No deletions were detected in any sample examined; whole fish n = 5, heads only n = 6, hearts only n = 3.
(TIF)

Figure S3 **Phenotypic analysis of SB injected embryos.** Embryos and larvae were examined at various time points for phenotype. As with TB-injected embryos, SB-injected embryos develop an area of increased density in the head at 24 hpf (arrow). At 48 hpf, SB-morphant embryos have pooled blood below the heart and hindbrain ventricle enlargement (arrow). By 72 hpf, morphant fish develop enlarged pericardium and have edema around the eyes by 96 hpf. All of these observations are consistent with those observed in TB-injected fish (Fig 2).
(TIF)

Figure S4 **Histological examination of MMC (A, C) and TB (B, D) morphant fish.** All sections are displayed with anterior regions to the left. Larval fish were oriented laterally in agarose molds before being embedded in paraffin and sectioned sagittally in 4 µm sections. Sections of larval heads (A, C) are shown at 48 hpf. Enlarged sections of larval eyes (B, D) are shown at 72 hpf. The retinal ganglion cell layer (RGC) is noted with yellow bracket.
(TIF)

Movie S1 **Startle response of MMC morphants at 72 hpf.**
(MOV)

Movie S2 **Startle response of Opa1 morphants at 72 hpf.**
(MOV)
