# Supplementary figures and images for: Opa1 Is Required for Proper Mitochondrial Metabolism in Early Development
Source: PLoS One. 2013 Mar 14;8(3):e59218. doi: 10.1371/journal.pone.0059218 (PMC3597633; doi:10.1371/journal.pone.0059218)

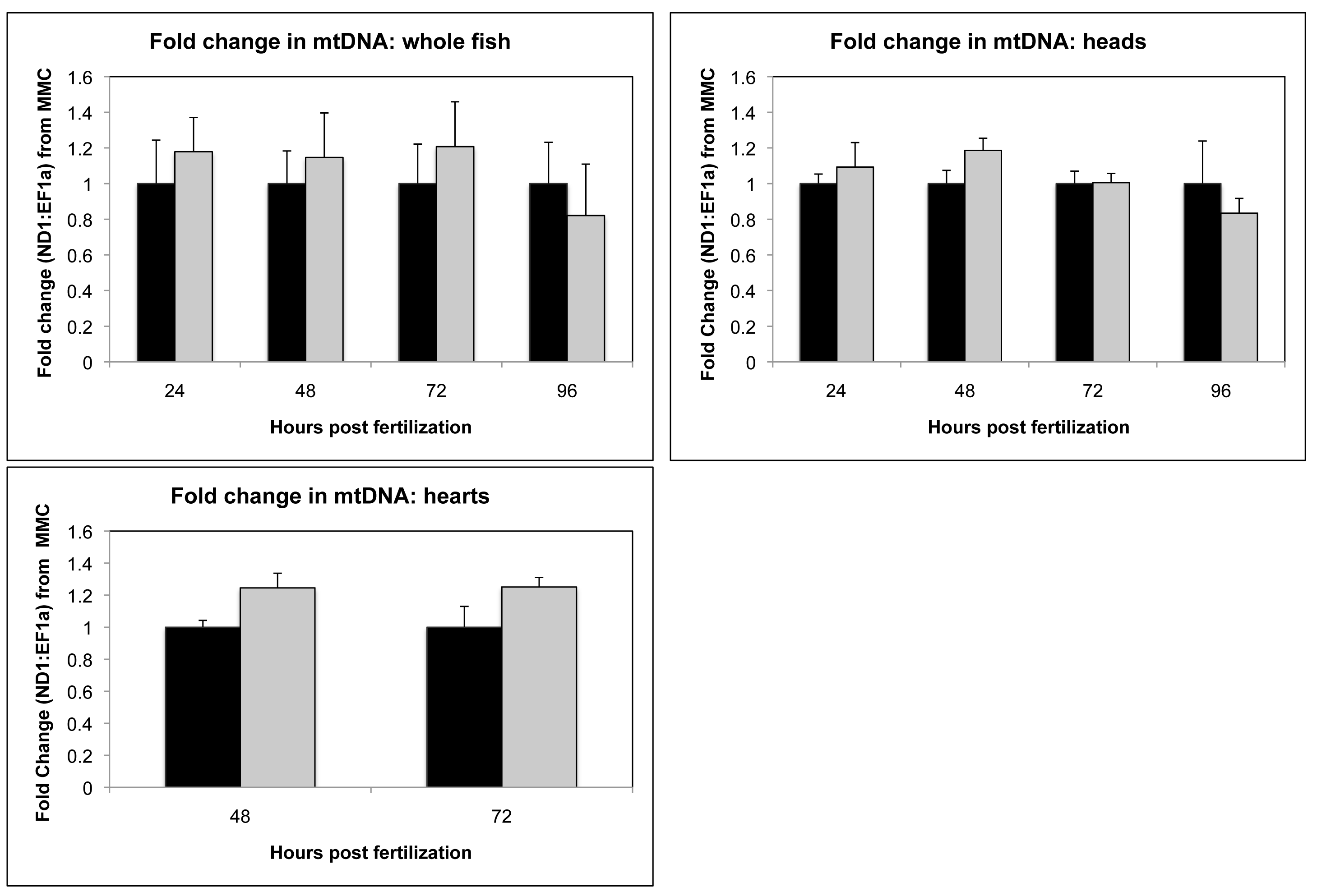

Supplement: Figure S1 — MtDNA content analysis in MMC morphants (black) and Opa1 morphants (grey). Whole fish, heads or hearts only were pooled from 10, 20 or 20 larvae respectively and DNA analyzed by QPCR for mtDNA:nuclear DNA ratio. Multiple independent injection experiments were performed for each data set. Fold change was calculated using the 2∧ΔΔCt method comparing Ct values obtained from nd1 (mtDNA-encoded gene) and ef1a (nuclear single copy gene). Error bars show SEM. N = 5 for whole fish, n = 6 for heads, n = 3 for hearts. No significant differences were obtained. (TIF) [file pone.0059218.s001.tif]

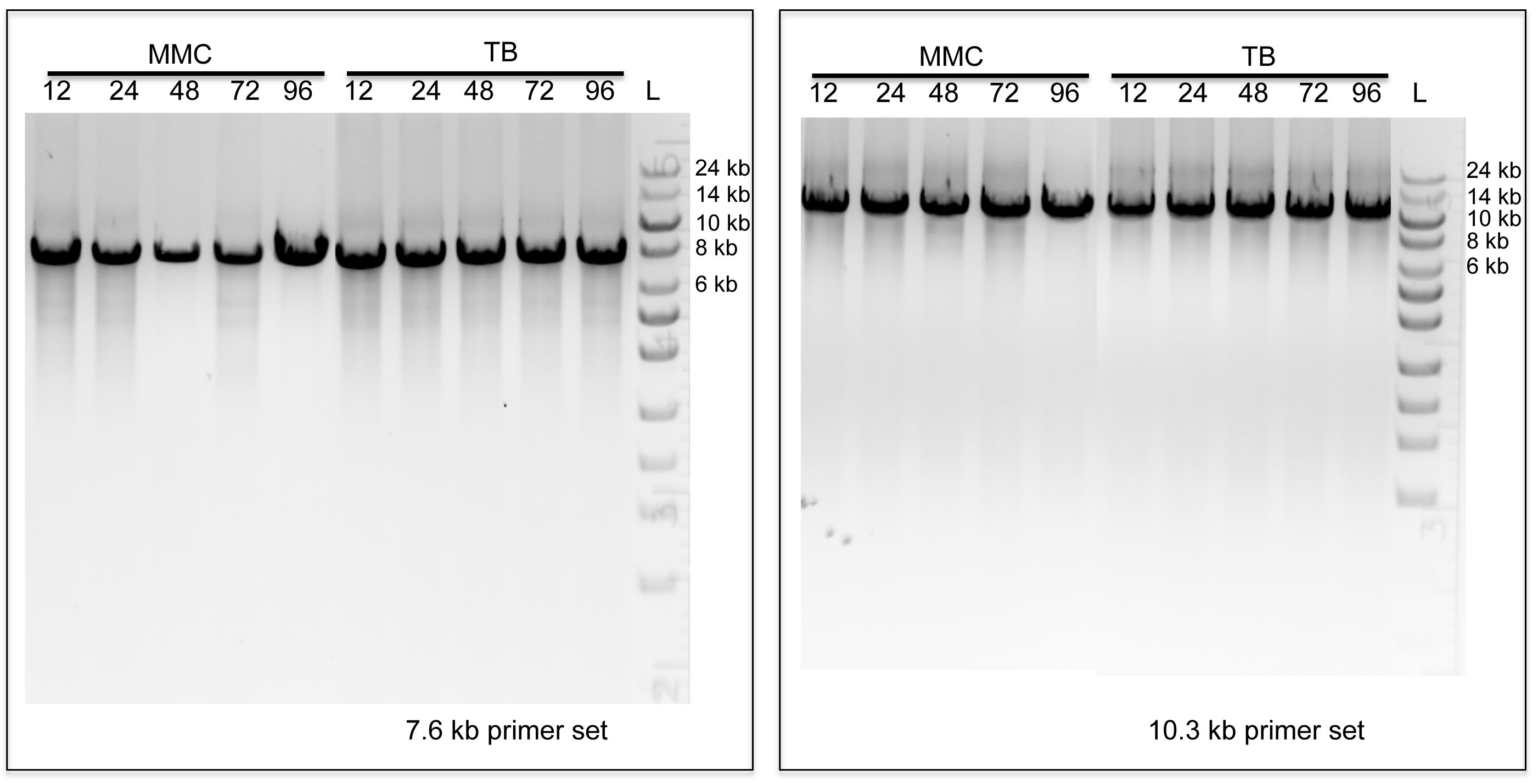

Supplement: Figure S2 — MtDNA deletion analysis. DNA was extracted from whole fish, heads or hearts at various time points (12, 24, 48, 72 and 96 hpf) and analyzed by XL-PCR using two sets of primers, one amplifying a 7.6 kb region of mtDNA and the other a 10.3 kb region of mtDNA. PCR products were analyzed by electrophoresis on 0.8% agarose gels. Representative gels are shown from whole fish samples. No deletions were detected in any sample examined; whole fish n = 5, heads only n = 6, hearts only n = 3. (TIF) [file pone.0059218.s002.tif]

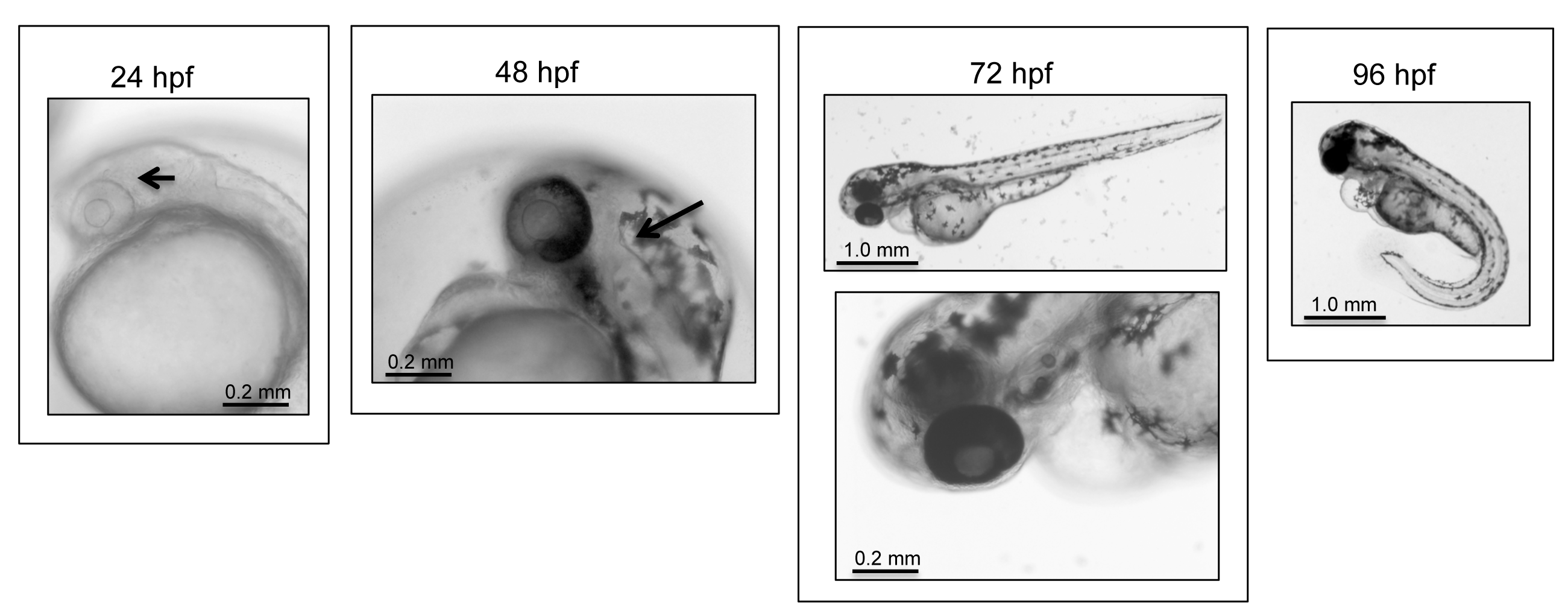

Supplement: Figure S3 — Phenotypic analysis of SB injected embryos. Embryos and larvae were examined at various time points for phenotype. As with TB-injected embryos, SB-injected embryos develop an area of increased density in the head at 24 hpf (arrow). At 48 hpf, SB-morphant embryos have pooled blood below the heart and hindbrain ventricle enlargement (arrow). By 72 hpf, morphant fish develop enlarged pericardium and have edema around the eyes by 96 hpf. All of these observations are consistent with those observed in TB-injected fish (Fig 2). (TIF) [file pone.0059218.s003.tif]

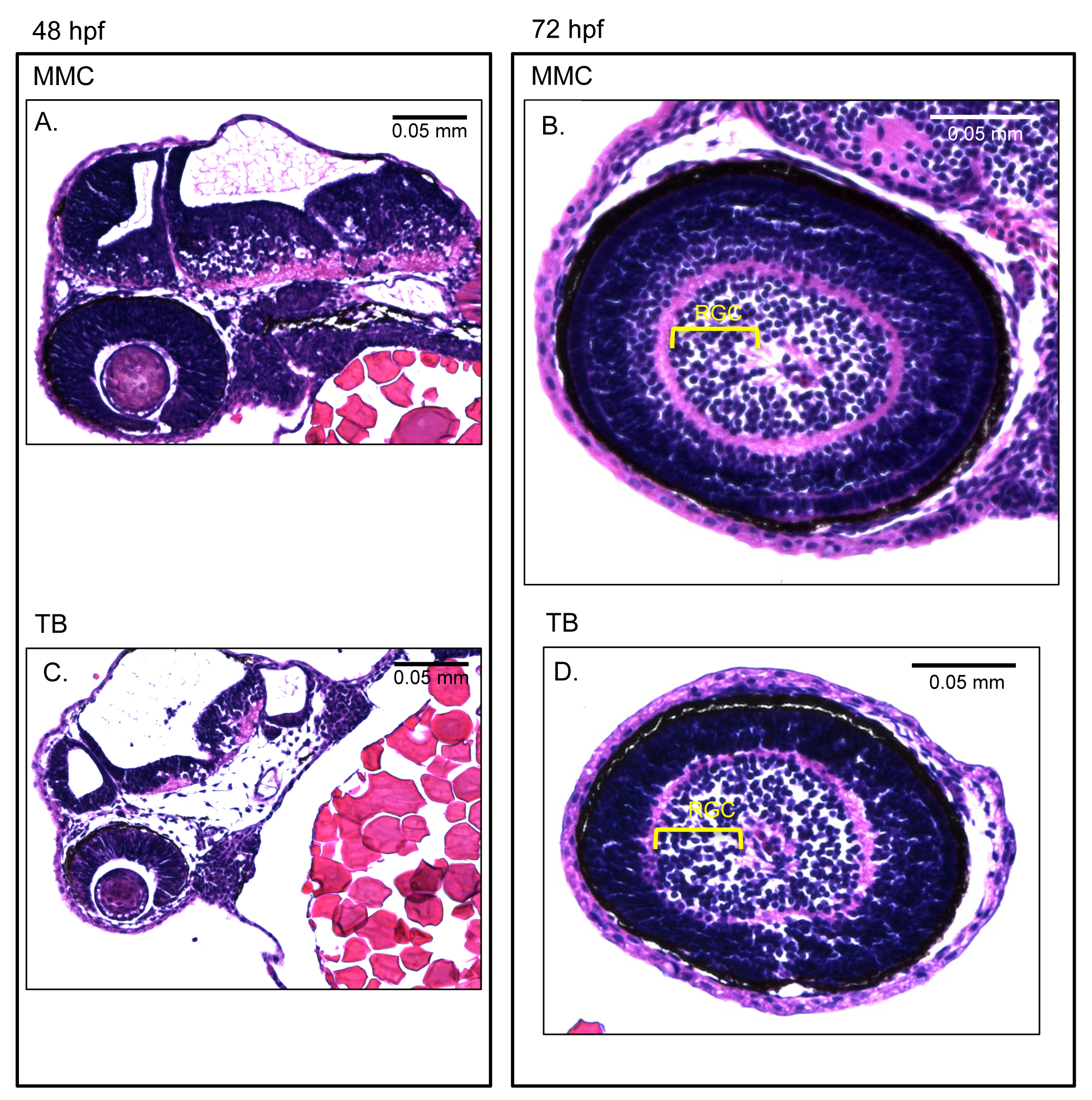

Supplement: Figure S4 — Histological examination of MMC (A, C) and TB (B, D) morphant fish. All sections are displayed with anterior regions to the left. Larval fish were oriented laterally in agarose molds before being embedded in paraffin and sectioned sagittally in 4 µm sections. Sections of larval heads (A, C) are shown at 48 hpf. Enlarged sections of larval eyes (B, D) are shown at 72 hpf. The retinal ganglion cell layer (RGC) is noted with yellow bracket. (TIF) [file pone.0059218.s004.tif]
